# Mental Health Problems and Sociodemographic Correlates in Elderly Medical Inpatients in a University Hospital in Egypt

**DOI:** 10.1155/2013/923710

**Published:** 2013-08-29

**Authors:** Emam M. Esmayel, Mohsen M. Eldarawy, Mohamed M. Hassan, Amira A. Mahmoud, Salem Y. Mohamed

**Affiliations:** Internal Medicine Department, Zagazig Faculty of Medicine, Zagazig 44519, Egypt

## Abstract

*Background*. Depression and cognitive impairment are two common mental and public health problems especially among elderly. In this study, we determined the prevalence of these problems and their associations with sociodemographic factors among hospitalized elderly in Egypt. To achieve this, 200 elderly medical inpatients were included in this cross-sectional study. *Methods*. Comprehensive geriatric assessment was done for every participant. Sociodemographic variables were assessed by interviews with patients and their family members. Depressive symptoms were screened for by the 15-item Geriatric Depression Scale (GDS), and the presence of depressive symptoms was defined as a GDS score of ≥6. Cognitive impairment was assessed by the Mini-Mental State Examination (MMSE) Scale, and cognitive impairment was defined as a MMSE score of ≤23 out of a total score of 30. *Results*. The prevalence of both depressive symptoms and cognitive impairment was 72% and 30%, respectively. Significant associations were noticed between each of depressive symptoms and cognitive impairment, and low income and advancing age (*P* < 0.01), respectively. Other associations were insignificant. *Conclusions*. The findings of this study may be an alarm for health authorities and staffs involved in elderly care to increase their awareness of social and mental health problems among the elderly.

## 1. Introduction

 The aging of the world's population is a global phenomenon with extensive economic and social consequences [1], and the poor understanding of elderly life under changing economic and social norms has led to a weak care and support of them [2]. The rapid increase in the elderly population has engendered public concern about issues associated, such as successful aging and social factors [3]. Factors that affect social engagement in the elderly are various, including physical function, mental function, and socioeconomic issues [4].

Despite growing concern with the elderly population, little attention has focused on their mental health [5]. Depression and cognitive impairment (CI) are two common mental health problems among elderly [6]. Detecting these two health problems in medical inpatients is important as they are associated with limitations in physical and social functioning [7–9]. Also, the recognition of depression and CI is of importance because they may respond to treatment, thereby reducing some of the clinical complications in management of elderly [10]. However, these two conditions are often underdiagnosed in medical settings or simply dismissed as inevitable consequences of aging [11, 12].

 One of the main features of the Egyptian population over the last few decades is the gradual increase in the number of older people [13]. However, there has been a dearth of studies on geriatric mental health problems in Egypt.

 So, this work was constructed in order to screen for the prevalence of depressive symptoms and CI among hospitalized elderly in Internal Medicine wards, Zagazig University hospital, Zagazig, Egypt, and to study the associations between sociodemographic profile of patients and these two mental health problems.

## 2. Methods

 This was a cross-sectional study conducted in the Internal Medicine Department, Faculty of Medicine, Zagazig University, Zagazig, Egypt. The duration of this study was one year from April 2012 to March 2013.

 Two hundreds, randomly selected, elderly (age ≥ 65 years) medical inpatients were included. We first looked at medical charts and decided not to include patients with emergency conditions, history of mental illness, psychotropic drug intake, and communication problems, for fear of obtaining inaccurate results. Twenty-two patients were approached for consent but refused or were unable to consent for various reasons (CI, illness, etc.) and were not included among the 200 participants. All the 200 included participants gave informed consent and the study had approval from local ethical committee.

A comprehensive geriatric assessment was done for each participant including full history intake, complete clinical examination, and assessment of sociodemographic characteristics through personal interviews with the patients and family members. Sociodemographic variables included inquiry about age, gender, residency, level of education, income, marital status, presence of offspring, and doing exercises.

 Screening for depression was done using the 15-items Geriatric Depression Scale (GDS) which has shown good sensitivity and specificity for predicting depression in different settings. It consists of 15 questions in a yes/no format, with total scores that ranged from 0 to 15 [14]. A cutoff value of ≥6 was used to predict depression and to compare the data with other studies.

 Assessment of cognition was done using the Mini-Mental State Examination (MMSE) Scale which is one of the most commonly used global cognitive screening measures because it is quick and easy to administer. It includes specific questions related to attention, orientation, memory, calculation, and language. The test score is based on 30 total points, and CI is indicated by a score of ≤23 [15].

### 2.1. Statistical Analysis

Statistical package for social sciences (SPSS) version 10 was used for data entry and analysis. Descriptive statistics were used to present distribution of study population. Analysis of variance (ANOVA) and Chi square (*χ* ^2^) were used to examine significant associations between variables. A *P*  value < 0.05 was considered statistically significant.

## 3. Results

The study included 200 participants (Table 1), 112 males (56%) and 88 females (44%). Most of the participants came from rural areas (*n* = 168, 84%) and 168 (84%) were illiterate. According to age, 164 (82%) had ages that ranged from 65 to <75 years (young old), 28 (14%) had ages that ranged from 75 to <85 years, and 8 (4%) were above 85 years old. Financially, incomes did not suffice 112 (56%) of the participants. Interestingly, no participant was performing physical exercises.

Table 2 shows the prevalence of depressive symptoms and CI as indicated by the GDS and MMSE, respectively. More than two-thirds (*n* = 144, 72%) of the participants were screened positive for depressive symptoms, and nearly one-third (*n* = 60, 30%) had cognitive impairment.

There was significant association between MMSE scores and age, while GDS scores insignificantly associated with age. Meanwhile, GDS scores were significantly associated with income, while MMSE scores were not (Table 3). 

No significant association was noticed between either GDS or MMSE scores and both gender, education, residence, and marital status. Approximately 40 (28.5%) of people that screened positive for depressive symptoms also had evidence of CI and they represented two-thirds of total patients with CI.

## 4. Discussion

 Data in this cross-sectional study are not general epidemiologic data but rather data on hospitalized inpatients. The prevalence of depressive symptoms among 200 elderly medical inpatients was 72% according to GDS. Prevalence rates of depression showed wide variability in studies that assessed it among elderly [16]. These differences may be due to the differences between communities. Also, these differences can be explained by the allocation of subjects, variety of instruments, staff (psychiatrics and nonpsychiatrics), and type of sample.

 The high rate of prevalence of depressive symptoms noticed in this study was also noticed in some Egyptian studies. Hamza et al. [17] reported a rate of depression of 64% among 100 elderly patients recruited from the inpatient unit, Ain Shams University hospitals, a result that is near to ours.

 On the other hand, Shehata et al. [18] reported a prevalence rate of depression of 31.4% among 86 elderly subjects in suburban community in Egypt, while Eman Mohamed and Mohamed Abd-Elhamid [13] found a prevalence of 25.3% among 466 elderly subjects attending geriatric clubs in Assiut city, Egypt.

 The higher rates of depressive symptoms observed in our study and that of Hamza et al. [17] may be explained that the participant elderly in both studies were hospitalized patients, while the participants in the other two studies [13, 18] were free-living patients. Higher rates of depression were observed in hospitalized elderly versus community-dwelling elderly [5]. Adding to the evidence of this observation, Hadi et al. [19] found that 53% of their cases (hospitalized inpatients in Internal and Surgical wards) were borderline cases of depression and 42% were probable cases of depression. Also, a very recent study in Alexandria reported higher prevalence of depression among hospitalized elderly (79%) compared to institutionalized (36%) and community-dwelling (24%) elderly [20]. Higher rates of depression among hospitalized inpatients may be explained that the patients are sick and may be depressed by being ill, being away from their families, being unable to work, and facing a risk of death.

 In this study, the prevalence rate of cognitive impairment was 30%, a result that is near to that obtained by Hamza et al. [17] who also used the MMSE scale. Shehata et al. [18] reported also a near prevalence rate (26.7%).

 On the other hand, Shawky Khater and Fawzy Abouelezz [21] identified CI in 38.3% of their 120 elderly living in elderly homes. Also, Joray et al. [22] reported a prevalence rate of 32% out of 401 elderly medical inpatients, and Buurman et al. [23] reported high prevalence rate of CI (40%) among acutely hospitalized older patients.

 As in case of depression, prevalence rates of cognitive impairment also vary in different studies all over the world which may be due to the same causes as in case of depression.

 In this study, significant association was noticed between depressive symptoms and low income, while no significant association was noticed between depressive symptoms and other sociodemographic variables. Various associations between depression and sociodemographic variables were noticed in various studies. Hamza et al. [17] did not notice any significant association between depression and sociodemographic variables, while Sidik et al. [1] found significant association with income (as in our study). Also, Shehata et al. [18] found significant association with gender only, while Hadi et al. [19] found significant associations with age and education. This variation in the associations of depression with sociodemographic variables may be due to the same causes of variation in prevalence rates mentioned earlier.

 As regards cognitive impairment associations in this study, significant association was noticed only with age, a result that is similar to that obtained by Ortiz et al. [16]. Also, Chen et al. [24] found that cognitive changes in hospitalized elderly were predicted by age.

 It is worthy to note that different studies reported contradictory associations between CI and sociodemographic variables. Sidik et al. [1] found significant association between CI and living alone, while Ortiz et al. [16] reported the reverse. Also, while we found insignificant association with educational level, both Sidik et al. [1] and Ortiz et al. [16] found significant association between CI and illiteracy.

 A possible limitation to this study is the high prevalence of illiteracy (84%) and the lack of distinction between levels of educational attainment which might affect MMSE scores. Although Dimitrov et al. [25] found in a Bulgarian population that lower education was associated with poorer MMSE performance but not with higher prevalence of dementia [25]; it might be better to use a different measure of cognitive function in future studies.

 The differences between this study and studies from elsewhere in the associations between different mental health problems and sociodemographic variables suggest that some of these associations may depend on cultural context.

 It is also worthy to note that no participant in this study was performing any physical exercise, and whether there is a relation between exercise and mental health requires future studies to elucidate.

## 5. Conclusions and Recommendations

 In this study, mental health problems, namely, depressive symptoms and cognitive impairment, were highly prevalent (72% and 30%, resp.) among elderly medical inpatients. They were also significantly associated with some sociodemographic variables (low income and old age, resp.). Psychosocial intervention, counseling, and psychiatric consultation are needed for inpatients whose screening results were positive. Our findings may be an alarm for health service authorities to increase their awareness of these social and mental health problems. It is also important for physicians and staffs concerned with the wellbeing of elderly subjects to recognize the impact of social and mental health dimensions. Our findings may be used as a baseline for larger in-depth studies to identify factors associated with these problems and to develop community-based health programs that screen for, and accurately deal with, social and mental dimensions.

## Figures and Tables

**Table 1:** Sociodemographic characteristics of patients included in this study.

	Number	%
Young old (65–<75 years)	164	82
Old old (75–<85 years)	28	14
Oldest old (≥85 years)	8	4
Male gender	112	56
Rural residency	168	84
Illiteracy	168	84
Low income	112	56
Married	80	40
Having offspring	192	96
Doing exercises	0	0.00

**Table 2:** Depression and cognitive impairment as indicated by the Geriatric Depression Scale (GDS) and Mini-Mental State Examination (MMSE) Scale, respectively.

	Number	%
GDS score < 6 (normal)	56	28
GDS score ≥ 6 (screened positive for depression)	144	72
MMSE score > 23 (normal)	140	70
MMSE score ≤ 23 (cognitive impairment)	60	30

**Table 3:** Association between Geriatric Depression Scale (GDS) and Mini-Mental State Examination (MMSE) Scale with age and income.

	GDS (mean ± SD)	MMSE (mean ± SD)
Young old (65–<75 years) (*n* = 164)	7.85 ± 3.3	19.7 ± 6.2
Old old (75–<85 years) (*n* = 28)	9.6 ± 4.1	13.7 ± 7.7
Oldest old (≥85 years) (*n* = 8)	9 ± 4.2	16.5 ± 10.6
*F* value	1.67	5.42
*P*	0.24	0.008*
Low income (*n* = 112)	8.9 ± 3	17.8 ± 6.7
Moderate income (*n* = 88)	7.1 ± 3.8	19.9 ± 6.6
*χ* ^2^	2.63	1.57
*P*	0.009*	0.11

*Significant *P*, *n* = number.
